# Editorial: Climate change, air pollution, and health inequality: vulnerability of marginalized populations

**DOI:** 10.3389/fpubh.2026.1957713

**Published:** 2026-09-17

**Authors:** Mohd Faiz Ibrahim, Nurzawani Md Sofwan, Arthit Phosri, Chris Fook Sheng Ng, Siti Aisah Mokhtar

**Affiliations:** 1 Environmental Health Research Center, Institute for Medical Research, National Institutes of Health, Ministry of Health Malaysia, Shah Alam, Malaysia; 2 Atmospheric Environment and Air Quality Research Initiative Group, Faculty of Health Sciences, Universiti Teknologi MARA, Sarawak Branch, Kota Samarahan, Sarawak, Malaysia; 3 Department of Environmental Health Sciences, Faculty of Public Health, Mahidol University, Bangkok, Thailand; 4 Department of Global Health Policy, Graduate School of Medicine, The University of Tokyo, Tokyo, Japan; 5 Department of Community Health, Faculty of Medicine and Health Sciences, Universiti Putra Malaysia, Serdang, Malaysia

**Keywords:** disease burden, effect modification, environmental epidemiology, environmental justice, exposure assessment

## Introduction

Climate change and air pollution are no longer separate environmental challenges. Driven by common anthropogenic processes, climate change and air pollution interact across atmospheric, ecological, and social systems to influence human health in ways that are increasingly difficult to disentangle (1). Rising temperatures alter the formation and distribution of air pollutants, while changing weather patterns, wildfires, and dust storms further modify population exposures and amplify health risks (2–4). These environmental pressures occur alongside demographic transitions, rapid urban growth, and widening social inequities, creating a complex web of interacting stressors that disproportionately affect marginalized populations. These include low-income communities, migrant workers, older adults, and geographically isolated populations, whose adaptive capacity is often constrained by social, economic, and structural disadvantages (5, 6). Collectively, these interconnected environmental and social pressures represent one of the defining public health challenges of the 21st century.

The studies included in this Research Topic reflect the evolving direction of environmental health research. Spanning diverse geographical settings and employing approaches ranging from time-series analyses and retrospective cohort studies to burden-of-disease assessments, policy evaluations, vulnerability assessments, and behavioral research, the 31 articles move beyond documenting individual exposure–response relationships. Together, they illustrate how environmental risks are shaped by the interaction between climatic change, air pollution, socioeconomic conditions, and population vulnerability, while highlighting opportunities for adaptation, mitigation, and more equitable public health action.

Across this Research Topic, a common message emerges: understanding the health impacts of climate change and air pollution requires moving beyond single exposures and individual diseases toward integrated perspectives that recognize the interplay between environmental change, social inequity, and population resilience. This Research Topic brings together these different strands of evidence to provide a broader perspective on the future direction of environmental health research. The discussion that follows draws together these shared themes and considers their implications for environmental epidemiology, public health policy, and equitable climate action among marginalized populations.

## Converging environmental stressors

One of the clearest messages emerging from this Research Topic is that climate change and air pollution cannot be adequately addressed as independent environmental health challenges. Although traditionally investigated as distinct risk factors, the studies collectively illustrate that these exposures are interconnected through shared drivers, overlapping exposure pathways, and common biological mechanisms. Rapid urbanization, industrialization, fossil fuel combustion, and land-use change simultaneously accelerate climate change while increasing ambient and household air pollution, creating environmental conditions in which populations are exposed to multiple hazards rather than isolated risks. Across diverse geographical settings—from rapidly urbanizing cities to rural and resource-limited communities—the evidence consistently suggests that environmental exposures are becoming increasingly complex, dynamic, and interdependent, emphasizing the need for integrated approaches to environmental health research and policy.

This convergence is evident in the diversity of environmental stressors represented across the Research Topic. Wang Y. et al. examine ambient particulate and gaseous pollutants in relation to cardiovascular morbidity in China, reflecting the continuing importance of urban air pollution. The Research Topic extends beyond conventional ambient exposures. Shen et al. demonstrate the health consequences of sand and dust events, while Solomon et al. examine wildfire smoke, PM_2.5_ and extreme heat among low-income agricultural-worker households, illustrating how pollution and climatic hazards can occur together. The settings in which exposure occurs are equally diverse. Feng et al. compare the health burden attributable to ambient and household air pollution across 10 populous countries, while Mohamed et al. draw attention to volatile organic compounds and combustion emissions in indoor environments. From a climatic perspective, Guo et al. examine the cardiovascular burden associated with high and low non-optimal temperatures, and Zhang and Li synthesize evidence on the influence of weather conditions on wellbeing and emotion. Together, these contributions illustrate an environmental health context that extends across ambient and indoor environments, pollution sources and climatic conditions, with populations experiencing different combinations of stressors across place and time.

Another important theme is the recognition that environmental risks are rarely immediate or linear. Wang Y. et al. demonstrate lagged and non-linear associations between ambient air pollutants and cardiovascular outpatient visits, while Du et al. report similar temporal and non-linear patterns for air pollution and thyroid goiter admissions. Chen et al. further show that short-term associations between multiple ambient pollutants and non-accidental mortality vary across lag periods. Beyond these individual exposure–response patterns, several contributions consider how environmental conditions may interact. Han et al. examine the interplay between meteorological factors and particulate pollution in relation to coronary heart disease hospitalizations, while Wang W. et al. assess the combined influence of temperature, heatwaves, diurnal temperature range and oxidative air pollutants on circulatory mortality. Together, these studies suggest that environmental health risks are shaped not only by the level of exposure, but also by its timing, co-occurrence with other stressors, and prevailing meteorological conditions. Such complexity supports epidemiological approaches that can account for non-linearity, delayed effects and interactions rather than treating environmental hazards as independent and static exposures.

## Unequal vulnerability

Perhaps the most compelling narrative emerging from this Research Topic is that environmental exposures do not translate into health outcomes uniformly across populations. Although climate change and air pollution are global phenomena, their health consequences are profoundly shaped by the social, economic, demographic, and environmental contexts in which people live. Across diverse geographical settings, the studies consistently demonstrate that vulnerability is not determined solely by the magnitude of environmental exposure but by the interaction between environmental exposure, biological susceptibility, and the adaptive capacity to anticipate, cope with, and recover from environmental hazards. This perspective moves environmental epidemiology beyond identifying environmental risks toward understanding the mechanisms by which those risks lead to health inequalities.

The Research Topic illustrates that vulnerability operates across multiple and often overlapping dimensions. Chen et al. identify clear differences in pollution-related mortality across population subgroups, with older adults showing increased susceptibility across all pollutants and sex-specific patterns observed for individual pollutants, including greater susceptibility to SO_2_ among women and to PM_2.5_ among men. Shen et al. report greater susceptibility to respiratory mortality during sand and dust events among older adults and women, while Lian et al. similarly identify variation in the effects of long-term ozone exposure on chronic obstructive pulmonary disease (COPD) exacerbations according to demographic and clinical characteristics. Yet, vulnerability extends beyond biological susceptibility. Liang et al. show how the health vulnerability of migrant workers under different seasonal temperatures varies across generations and is intertwined with socioeconomic and health-system conditions. Wangsan et al., from a different perspective, demonstrate how people modify physical activity and air-quality monitoring behaviors during periods of elevated PM_2.5_ Together, these contributions show that vulnerability emerges from the interaction of biological susceptibility with social position, living and working conditions, and the capacity to respond to environmental risk.

Place is another important dimension of this vulnerability. Nazakat et al. address this directly by developing and validating a household climate-change vulnerability assessment tool for small-island communities in Malaysia, incorporating exposure, sensitivity, and adaptive capacity into a context-specific measure. Solomon et al. provide a different example among low-income agricultural-worker households in California, USA, who are exposed to wildfire smoke, PM_2.5_, and extreme heat, where environmental hazards intersect with socioeconomic and occupational circumstances. Shahbaz et al. similarly examine respiratory health among communities living near industrial areas in Karachi, Pakistan, illustrating how residential location can shape environmental exposure. These studies reinforce that vulnerability is not simply a characteristic of individuals; it is influenced by the places in which people live and work and by the resources available within those settings.

The global burden studies add another dimension by showing how environmental inequalities are distributed across countries and levels of socioeconomic development. Feng et al. document substantial differences in disease burden attributable to ambient and household air pollution across 10 populous countries, while Xia et al. show that the declining global stroke burden attributable to household air pollution from solid fuels remains unequally distributed. Guo et al. similarly identify marked regional and socioeconomic heterogeneity in cardiovascular burden attributable to non-optimal temperatures. Hodgson et al. bring this question of inequality into the policy setting, showing that different PM_2.5_ reduction strategies may produce different population-exposure and equity outcomes. Together, these studies caution against interpreting improvements in average exposure or age-standardized disease burden as evidence that environmental inequalities have been resolved.

Collectively, the studies reinforce the role of environmental justice as a central consideration in contemporary environmental epidemiology. Vulnerability should not be understood as an intrinsic characteristic of particular populations, but as the product of unequal exposure, susceptibility and capacity to adapt. Reducing the health impacts of climate change and air pollution therefore requires attention not only to environmental hazards themselves, but also to the social and structural conditions that determine who is most exposed, who experiences the greatest health consequences, and who has the resources to respond.

## Environmental epidemiology in transition

Beyond the individual findings, this Research Topic reflects the continuing evolution of environmental epidemiology as a discipline. Traditionally, much of environmental epidemiology has focused on quantifying associations between individual environmental exposures and specific health outcomes. The studies in this Research Topic retain this epidemiological foundation but extend it through more complex exposure–response analyses, broader health outcomes, assessments of population burden and inequality, and approaches that consider vulnerability, behavior, and policy. Collectively, they illustrate a field moving beyond identifying whether an environmental hazard affects health toward understanding when, where, and among whom its effects are greatest.

This transition is evident in the breadth of health outcomes examined across the Research Topic. Cardiovascular and respiratory health remain prominent, with Wang Y. et al. examining air pollution and cardiovascular outpatient visits, Lian et al. assessing long-term ozone exposure and COPD exacerbations and Wu X. et al. examining long-term PM_2.5_ exposure and new-onset lung disease among middle-aged and older adults. Other studies extend environmental epidemiology into less commonly examined outcomes. Du et al. investigate air pollution in relation to thyroid goiter admissions, Wu Z. et al. examine short-term air pollution and gastrointestinal cancer mortality, and Li et al. assess PM_2.5_ constituents in relation to glaucoma. Rho et al. examine long-term PM10 exposure in relation to prostate cancer, while Wang L. et al. synthesize pathways linking climate change with skin cancer risk. Zhang and Li extend the scope further by synthesizing evidence on weather conditions, wellbeing and emotion. Together, these contributions illustrate that the health consequences of environmental change are being examined across an increasingly broad range of physiological, functional and wellbeing outcomes.

The methodological approaches represented in the Research Topic have expanded alongside this broader scope. Time-series and case-crossover studies remain valuable for characterizing short-term associations, lag structures and non-linear exposure–response relationships, as demonstrated by Chen et al., Shen et al., Wang Y. et al., and Han et al. At a different scale, Wang and Lai, Feng et al., Xia et al., Wu E. et al., Wu et al., and Guo et al. use burden-of-disease approaches to examine temporal trends, demographic patterns and inequalities across countries and levels of socioeconomic development. Nie et al. extend this population-level perspective through an analysis of global disparities in non-alcoholic steatohepatitis (NASH)-associated liver cancer, showing how population growth, ageing, and epidemiological changes contribute to changing mortality patterns across sociodemographic settings. These population-level analyses complement conventional measures of association by showing how environmental risks translate into mortality and disability across large populations and how those burdens change over time. Given the diversity of approaches represented in the Research Topic, differences in exposure assessment, study design, spatial resolution, and outcome definitions should be considered when comparing findings across studies.

Other contributions extend the role of epidemiology beyond estimating disease occurrence. Nazakat et al. develop and validate a household climate-change vulnerability assessment tool, providing a means of characterizing exposure, sensitivity and adaptive capacity before health consequences occur. Wangsan et al. examine behavioral responses to elevated PM_2.5_, while Sierpiński et al. explore differences in the adoption of personal measures to reduce air-pollution exposure. Zhao et al. approach the issue from a policy perspective by evaluating the health and economic implications of energy-conservation and emission-reduction measures. Together, these studies broaden the questions addressed by environmental health research from identifying environmental risks toward understanding vulnerability, behavioral responses and the potential consequences of intervention.

Taken together, the Research Topic reflects an environmental epidemiology that is becoming broader in both its questions and its applications. Estimating exposure–response relationships remains fundamental, but it increasingly sits alongside the assessment of disease burden, inequalities, vulnerability, behavior and policy. This wider perspective is particularly relevant as climate change and air pollution interact with demographic and socioeconomic transitions to create environmental health challenges that cannot be adequately understood through a single exposure, outcome or disciplinary lens, underscoring the need for greater multidisciplinary collaboration. The next challenge is to translate this expanding evidence base into public health decisions that reduce exposure, strengthen resilience and protect populations facing the greatest environmental risks.

## From evidence to action

The value of environmental epidemiology lies not only in identifying health risks but also in informing actions that reduce exposure and protect populations at greatest risk. Across this Research Topic, several contributions move in this direction by linking evidence on environmental hazards with mitigation, adaptation, behavioral responses, and policy. Together, they suggest that effective action requires more than a uniform reduction in environmental exposure; interventions must account for differences in pollution sources, local environmental conditions, population vulnerability, and the capacity of communities to respond.

At the environmental and policy level, several studies illustrate how epidemiological and exposure evidence can inform more targeted interventions. Jing and Xiaofeng identify the contributions of sand and dust events, heating, transport, industrial activity, and economic development to air-pollution patterns in Urumqi, providing a basis for source-specific pollution control. Hodgson et al. take this further by comparing PM_2.5_ policy scenarios in the West Midlands, showing that strategies focused on population exposure can generate broad health-equity benefits, although disadvantaged communities may remain disproportionately exposed. Zhao et al. approach mitigation from a health-economic perspective, evaluating the health benefits and economic costs of energy-conservation and emission-reduction policies. These studies illustrate the importance of connecting evidence on sources and exposures with policy choices and of assessing success not only through average environmental improvements but also through their distribution across populations.

Action also occurs at the level of communities and individuals. Nazakat et al. demonstrate how context-specific assessment of exposure, sensitivity, and adaptive capacity can help identify households vulnerable to climate-related hazards, providing a basis for more targeted preparedness and adaptation. Sierpiński et al. show that the adoption of personal measures to reduce air-pollution exposure varies according to financial and health circumstances, while Wangsan et al. document changes in physical activity, exercise location, and air-quality monitoring during periods of elevated PM_2.5_. Wang L. et al., through their review of climate change and skin cancer, similarly highlight opportunities for prevention through behavioral and environmental measures. These contributions indicate that adaptation cannot be separated from the social conditions that determine whether individuals and communities have the resources and opportunities to act.

The broader implication is that environmental health interventions should be judged not only by whether they reduce exposure or disease burden, but also by who benefits. Cleaner energy, emission controls, urban planning, and climate adaptation can improve population health, yet their benefits may remain uneven if existing social and geographic inequalities are overlooked (7, 8). Recent evidence further shows that climate mitigation can generate substantial public-health co-benefits, particularly through improvements in air quality, while the magnitude and distribution of these benefits may vary across populations and socioeconomic settings (9, 10). This broader perspective calls for environmental governance that considers not only environmental and health gains, but also how these benefits are distributed across populations and contribute to wider societal wellbeing. The evidence assembled in this Research Topic supports a public health response that combines environmental mitigation with targeted adaptation and attention to equity. For environmental epidemiology, this means extending the pathway from identifying associations to identifying susceptible populations, anticipating future burdens, evaluating interventions, and generating evidence to inform decisions at the local, national, and global levels.

## Conclusion

The studies in this Research Topic demonstrate that climate change, air pollution, and health inequality are closely interconnected public health challenges. Across diverse settings, the Research Topic shows that environmental risks are shaped by multiple and changing exposures, with their health consequences influenced by biological susceptibility, social conditions, and adaptive capacity. The breadth of methods and outcomes represented also reflects an environmental epidemiology that is expanding beyond individual exposure–outcome relationships toward understanding population burden, vulnerability, and opportunities for intervention. Moving forward, translating this evidence into effective mitigation and adaptation will require multidisciplinary public health actions that reduce environmental risks while prioritizing populations facing the greatest disadvantage. Equity should therefore be considered not only as a determinant of vulnerability, but also as an outcome against which the effectiveness of mitigation and adaptation strategies is evaluated.
